# The ecdysone receptor complex is essential for the reproductive success in the female desert locust, *Schistocerca gregaria*

**DOI:** 10.1038/s41598-018-36763-9

**Published:** 2019-01-09

**Authors:** Cynthia Lenaerts, Elisabeth Marchal, Paulien Peeters, Jozef Vanden Broeck

**Affiliations:** Molecular and Developmental Physiology and Signal Transduction research group, KU Leuven, Naamsestraat 59, P.O. Box 02465, B-3000 Leuven, Belgium

## Abstract

Ecdysteroid hormones influence the development and reproduction of arthropods by binding a heterodimeric complex of nuclear receptors, the ecdysone receptor (EcR) and the retinoid-X-receptor/ultraspiracle (RXR/USP). Here, we report on the *in vivo* role(s) of the ecdysone receptor complex, *SchgrEcR/SchgrRXR*, in the female reproductive physiology of a major phytophagous pest insect, i.e. the desert locust, *Schistocerca gregaria*. Tissue and temporal distribution profiles were analysed during the first gonadotrophic cycle of adult female locusts. RNA interference was used as a reverse genetics tool to investigate the *in vivo* role of the ecdysone receptor complex in ovarian maturation, oogenesis, fertility and fecundity. We discovered that silencing the ecdysone receptor complex in *S. gregaria* resulted in impaired ovulation and oviposition, indicative for a crucial role of this complex in chorion formation. We also found evidence for a feedback of *SchgrEcR/SchgrRXR* on juvenile hormone biosynthesis by the corpora allata. Furthermore, we observed a tissue-dependent effect of the *SchgrEcR/SchgrRXR* knockdown on the transcript levels of the insulin receptor and neuroparsin 3 and 4. The insulin receptor transcript levels were upregulated in the brain, but not the fat body and gonads. Neuroparsins 3 and 4 transcript levels were down regulated in the brain and fat body, but not in the gonads.

## Introduction

Ecdysteroids and juvenile hormones (JHs) are two major insect hormone families famous for their roles in development, moulting and metamorphosis. However, these hormones are also crucial in the reproductive physiology of insects, as reviewed by Raikhel *et al*.^1^. Both hormones act via a nuclear receptor, thereby regulating the transcription of several response genes. JH acts via the methoprene-tolerant (Met) receptor^2–7^, while the active ecdysteroid, 20-hydroxyecdysone (20E), acts via the heterodimeric ecdysone receptor complex, ecdysone receptor/ultraspiracle (EcR/USP)^8^. The hemimetabolan orthologue of USP is called retinoid-X-receptor (RXR). The crucial role of the ecdysone receptor complex during moulting of the desert locust, *Schistocerca gregaria*, was recently described^9^. The precursor for ecdysteroid synthesis is cholesterol, which is converted to ecdysone and its active metabolite 20E through a series of oxidation and hydroxylation steps. These enzymatic conversions are catalysed by cytochrome P450 enzymes encoded by the *Halloween* genes *spook* (*Spo*), *phantom* (*Phm*), *disembodied* (*Dib*), *shadow* (*Sad*) and *shade* (*Shd*), as reviewed by Niwa and Niwa^10^. In *Schistocerca gregaria*, the *Halloween* genes *spook* (*SchgrSpo*), *phantom* (*SchgrPhm*) and *shade* (*SchgrShd*) were previously characterised in fifth nymphal locusts^11,12^.

In addition to their regulatory role in insect moulting, ecdysteroids are necessary in different aspects of female reproductive physiology. In all insects, ecdysteroids are incorporated in the oocytes as a maternal source of ecdysteroids during embryogenesis^13^. Most knowledge about the involvement of ecdysteroids in the regulation of ovarian maturation and oogenesis in insects was obtained in three holometabolous insects: the silk moth, *Bombyx mori*, the yellow fever mosquito, *Aedes aegypti*, and the fruit fly, *Drosophila melanogaster*. In *D. melanogaster*, ecdysteroid signalling is needed first in repression, and later in activation of niche and primordial cell differentiation in the last larval stage^14^. Furthermore, progression of oogenesis at the onset of vitellogenesis requires ecdysteroid signalling^15^. Mutants of the *Halloween* genes*, Spo* and *Shd*, display arrested oogenesis at the initiation of vitellogenesis^16,17^. *Usp* mutants on the other hand were not affected in the progression of oogenesis at the onset of vitellogenesis, but USP is required for chorion formation at the end of oogenesis^18^. In *A. aegypti*, a blood meal triggers the release of ovary ecdysteroidogenic hormone by the brain, which then induces the ovarian production of ecdysteroids that subsequently stimulate the fat body to produce vitellogenins that are packaged into the oocytes^19–21^. Furthermore, ecdysteroids are also involved in formation of the vitelline envelope in the primary follicle at the end of oogenesis^22^. In *B. mori*, ecdysteroids regulate the development of ovaries during pupal and pharate adult stages^23^. Ecdysteroids from the PG play a role in ovarian maturation and oogenesis, while ecdysteroids produced by the follicle cells are exclusively incorporated in the oocytes and have no autocrine/paracrine or endocrine function in the regulation of oogenesis. High ecdysteroid titres are necessary for the early events of oogenesis (previtellogenic and vitellogenic stage), while a decline of the ecdysteroid titre is required for choriogenesis^24^. Research on the regulation of ovarian maturation and oogenesis in more primitive insect orders with panoistic ovaries has seriously lagged behind on that in Holometabola with meroistic ovaries, with only a few studies performed in the cockroach species, *Blattella germanica*, the German cockroach, and *Diploptera punctata*, the Pacific beetle cockroach^25–27^, and in the house cricket, *Acheta domesticus*^28^. In both Orthoptera and Dictyoptera, JH acts as the primary regulator of ovarian development and ecdysteroid titres significantly increase at the end of oogenesis^1,28–31^. Moreover, it has been shown in *B. germanica* that 20E can induce precocious choriogenesis and that ecdysteroid signalling is needed for the proliferation and function of the follicle cells and normal choriogenesis^26,27,32,33^. Also in the cockroach *Diploptera punctata*, it has been shown that ecdysteroid signalling is crucial for the termination of vitellogenesis and proper chorion formation at the end of the gonadotrophic cycle^25^. For extensive literature reviews on the role of ecdysteroids in ovarian maturation and oogenesis, the reader is referred to Bellés and Piulachs^34^ and Swevers and Iatrou^13^. In the migratory locust, *Locusta migratoria*, ovarian development and vitellogenin synthesis in the fat body are dependent on JH, while large amounts of ecdysteroids are synthesized at the end of the gonadotrophic cycle^35^. Most ecdysteroids produced by the follicle cells surrounding the oocytes are incorporated into the developing eggs, while a small amount is released in the haemolymph, showing a peak in ecdysteroid titre at the end of the gonadotrophic cycle^11,35,36^. The exact role of these circulating ecdysteroids is still unclear.

In this study, we investigated the role of ecdysteroid signalling in the female reproductive physiology of the desert locust*, S. gregaria*. This swarm-forming phytophagous pest insect is a serious threat to agricultural production in some of the world’s poorest countries. Non-specific chemical insecticides are still the preferred method to combat locust plagues, but unfortunately they have a negative impact on the environment and non-target organisms. Therefore, the search for new, more biorational strategies to control locusts is crucial. Furthermore, given the differences between species and the limited knowledge in more primitive insect orders having the ancestral, panoistic type of ovaries, our results are a valuable addition to the current knowledge about the hormonal regulation of ovarian development and oogenesis. We report an in-depth transcript profiling of the heterodimeric receptor components, *SchgrEcR* and *SchgrRXR*, during the first gonadotrophic cycle of *S. gregaria*. Ovary and oocyte growth can be divided into four stages^37^: (1) Immature stage: ovaries and oocytes are small and white; (2) Previtellogenic stage: ovaries grow and fat body is well developed; (3) Vitellogenic stage: basal oocytes incorporate vitellogenins, ovaries and fat body are large; (4) Choriogenic stage: fat body is small, oocytes reach their maximum length, chorion is formed and ovulation can occur. The exact time frame of maturation is dependent on the environmental and food conditions. However, in our laboratory conditions the locust ovaries will enter the vitellogenic stage between days 8 and 10 after adult emergence and they reach the choriogenic stage between days 12 and 18. Using RNA interference (RNAi), we have shown a crucial role for the ecdysone receptor in ovarian maturation, ovulation and oviposition. We also investigated the effect of silencing the ecdysone receptor complex cross-talk with other hormonal pathways, namely JH, insulin signalling and neuroparsins. More specifically, we investigated the effect of the *Schgr*EcR/*Schgr*RXR knockdown on the transcript levels of different genes of interests, belonging to the previously mentioned hormonal pathways.

## Materials and Methods

### Rearing of animals

The animals were reared as previously described by Lenaerts *et al*.^38^. In the described experiments, locusts were synchronized on the day of ecdysis into the fifth larval and adult stage. Different experimental groups (distinctly labelled) were kept together in the same cage. For the temporal distribution profiles of the genes of interest, adults were synchronized on the day of ecdysis to the adult stage and dissected every other day until they were 18 days old. Moreover, adults were staged during dissection according to oocyte length, to make sure female animals within the same pool had the same physiological age. Upon dissection, the length of the basal oocytes was measured under the binocular microscope using millimetre paper. Female adult locusts were found to start vitellogenesis around day 8, to commence mating on day 12 and finally deposited their eggs around day 18.

### Tissue collection

Tissues were collected as previously described by Lenaerts *et al*.^38^. Tissues for the tissue and temporal expression profiles of *SchgrEcR* and *SchgrRXR* were collected in three independent pools (10 animals/pool). For the RNA interference experiments, tissues were collected in five independent pools consisting of three animals each. Tissues were stored at −80 °C until further processing.

### RNA extraction and cDNA synthesis

Depending on the tissue, different RNA extraction methods were used. Brain, thoracic ganglia, suboesophageal ganglion, salivary gland, fat body, midgut, muscle, ovaries, testes and accessory glands were transferred to MagNA Lyser Green Beads Tubes (Roche) and homogenized using a MagNA Lyser instrument (1 min, 6500 rpm; Roche). Subsequently, total RNA was extracted from these tissue homogenates using the RNeasy Lipid Tissue Kit (Qiagen) according to the manufacturer’s protocol. A DNase treatment (RNase-Free DNase set, Qiagen) was performed to eliminate potential genomic DNA contamination. Because of the relatively small size of the prothoracic glands (PG), corpora allata (CA) and corpora cardiaca (CC) total RNA from these tissues was extracted using the RNAqueous-Micro Kit (Ambion) according to the manufacturer’s protocol. The manufacturer’s recommended DNase step was subsequently performed. Purity and concentration of the resulting RNA samples were checked using a Nanodrop spectrophotometer (Nanodrop ND-1000, Thermo Fisher Scientific, Inc.). For each RNA sample, cDNA was synthesized by reverse transcription of 500 ng of RNA with the PrimeScript™ RT reagent Kit (Perfect Real Time) (Takara, Invitrogen Life Technologies), using both random hexamer primers and oligo(dT) primers, according to the manufacturer’s protocol. The 10 µL reaction was diluted sixteen-fold with Milli-Q water (Millipore).

### Quantitative real-time PCR (qRT-PCR)

Primers used in the qRT-PCR profiling are given in Supplementary Table 1. Primer validation and subsequent qRT-PCR reactions were performed as previously described by Lenaerts *et al*.^38^. In brief, we used the Fast SYBR® Green Master Mix (Applied Biosystems) and a StepOne System (ABI Prism, Applied Biosystems). Amplification efficiency and specificity, as well as dissociation curves, were checked for validation of the primer pairs. The primers used in all experiments have an efficiency between 90% and 110%, they amplify only the target of interest and show only one melting peak in the dissociation curve. ‘No template control’ reactions confirmed there was no contamination. For each experiment suitable reference genes were selected from a pool of candidate reference genes by means of the geNorm software^39,40^. The relative transcript levels of each gene of interest (GOI) were calculated using the comparative Ct method (normalization against selected reference genes and relative to a calibrator sample)^39^. qRT-PCR was used to determine the tissue and temporal distributions of *SchgrEcR* and *SchgrRXR* in the adult stage. The cDNA samples were derived from adult female locusts, with exception of the male accessory glands and testes. The transcript levels of the GOIs were normalized to *β-actin* and *elongation factor 1α* (*EF1α*) transcript levels. Moreover, qRT-PCR was used to check the efficiency of the RNAi-mediated knockdown of the known ecdysone receptor complex *(SchgrEcR/SchgrRXR)* as well as to determine the effect of silencing these genes on the relative transcript levels of several GOIs. In these RNAi experiments the transcript levels of the GOIs were normalized to *CG13220* and α-*tubulin1A* transcript levels for the ovaries, *CG13220, ubiquitin conjugating enzyme 10 (Ubi)* and *ribosomal protein* 4*9* *(RP49)* transcript levels for the fat body, and *β-actin* and *EF1α* transcript levels for the CA/CC complex. GraphPad Prism 6 (GraphPad Software Inc.) was used to test the statistical significance of the observed differences for the RNAi experiments. Normalized relative quantities were log-transformed to allow the use of parametric statistical tests.

### RNA interference experiments

#### Production of dsRNA

dsRNA constructs for *SchgrEcR* and *SchgrRXR* were produced using the MEGAscript® RNAi Kit (Ambion) according to the manufacturer’s protocol and as previously described by Lenaerts *et al*.^38^. Primers used to produce the dsRNAs are given in Supplementary Table 2. The dsRNA construct against *Schgr*EcR targets a region of 250 bp, while the dsRNA against *Schgr*RXR targets a region of 253 bp. Using Blast, it was confirmed that the dsRNA constructs are specific against EcR and RXR. Moreover, the specificity of our dsRNA constructs against *Schgr*EcR and *Schgr*RXR was previously confirmed by testing a second set of dsRNA constructs targeting a different region of each gene of interest (GOI)^9^.

#### RNAi experiment

Virgin female locusts were injected with 400 ng (in 10 µL Ringer solution) of dsRNA against *SchgrEcR* and *SchgrRXR* or *GFP* (control) six days after moulting to the last nymphal stage, as well as one, five, nine and thirteen days after moulting to the adult stage. These female locusts were kept with males of the same age till day 8 in the adult stage, so the females mature more synchronously. As the males start to display copulation behaviour around day 10 of the adult stage, they were removed on day 8 of the adult stage to prevent copulation with the virgin female locusts. A first group of locusts (N = 15 to 20) was dissected 12 days after ecdysis to check the knockdown efficiency and the effect of the knockdown on ecdysteroid titres in the haemolymph, ecdysteroid levels in the ovaries, and the transcript levels of other genes of interest, as well as the effect on ovarian and oocyte growth and maturation. A second group of locusts (N = 12) was used to observe copulation behaviour and post-copulation events.

### Ecdysteroid measurements using an enzyme immunoassay (EIA)

Ecdysteroid titres and levels were measured using the enzyme immunoassay (EIA), as previously described by Lenaerts *et al*.^38^. Both ecdysteroid titres in the haemolymph, as well as ecdysteroid levels in the ovaries were measured in 12-day-old adult virgin female locusts (N = 15–20).

### Microscopy and histological analysis

Dissected ovaries were rinsed in locust Ringer solution. Images of the ovaries and ovarioles were obtained using a light microscope (Zeiss SteREO Discovery.V8) equipped with an AxioCam ICc3 camera using the AxioVision 4.7 (Carl Zeiss-Benelux). Oocyte sections were made according to Billen^41^. In short, the ovarioles were fixed in 2% glutaraldehyde in sodium cacodylate buffer and postfixed in 2% osmium tetroxide in the same buffer. Afterwards, samples were dehydrated in a graded acetone series and embedded in araldite. Semi-thin (1 µm) sections (Leica EM UC6 microtome) were stained with methylene blue and thionin. Images of the oocyte sections were obtained with a light microscope (Zeiss Axio Imager Z1) equipped with an AxioCam MRm camera (1388 × 1040 pixels) using the Imaging software program Zen 2012 (Blue Edition; Carl Zeiss-Benelux).

### Copulation behaviour and post-copulation effects

To investigate the possible effects of the RNAi-mediated knockdown of the ecdysone receptor complex *(SchgrEcR/SchgrRXR)* on the first display of copulation behaviour and fertility, the locusts were injected as described earlier (§’RNA interference experiments’). Copulation behaviour, fecundity and fertility were observed as previously described by Lenaerts *et al*.^38^. The males used for these copulation experiments were mature virgin males.

## Results

### Tissue specificity and developmental transcript profiles during the first gonadotrophic cycle

**Figure 1:**
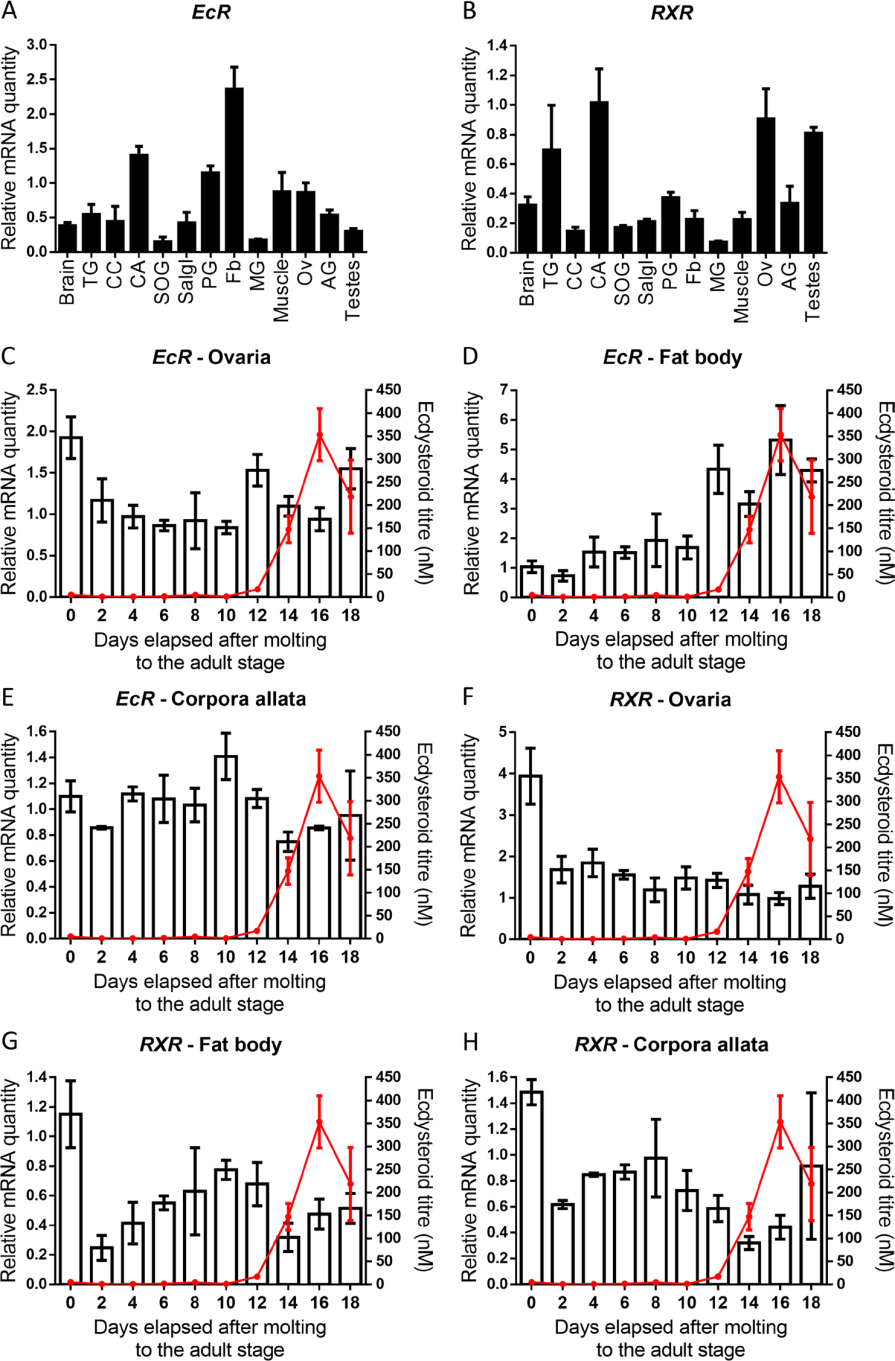
Tissue and temporal distribution of *SchgrEcR* and *SchgrRXR*. Relative transcript levels of (**A**) *SchgrEcR* and (**B**) *SchgrRXR* were measured in different tissues of adult locusts, using qRT-PCR. All tissues were dissected from 10-day-old adult female locusts, with exception of the accessory glands (AG) and the testes of 10-day-old adult male locusts. The data represent mean ± S.E.M. of three independent pools (40, 10 and 10 animals/pool), run in duplicate and normalized to *β-actin* and *elongation factor 1α* (*EF1α*) transcript levels. Other abbreviations X-axis: TG: thoracic ganglia; CC: corpora cardiaca; SOG: suboesophageal ganglion; Salgl: salivary gland; PG: prothoracic glands; Fb: fat body; MG: midgut; Ov: ovaries. Temporal distribution profile of **(C**–**E)** *SchgrEcR* and **(F**–**H)** *SchgrRXR* in the ovaries, fat body and corpora allata during the first reproductive cycle. Using qRT-PCR, relative transcript levels of *SchgrEcR* and *SchgrRXR* were measured every other day, starting on the day of moulting to the adult stage (day 0). Data represent mean ± S.E.M. (bars) of three independent pools of ten animals each, run in duplicate and normalized to *β-actin* and *EF1α* transcript levels. The ecdysteroid titre in the hemolymph (red line), expressed in nM, throughout the first reproductive cycle was measured with an EIA. The data represent mean ± S.E.M. of 6–18 haemolymph samples taken from different animals.

Using qRT-PCR, the tissue-specific and temporal distribution profiles of the components of the ecdysone receptor complex, *SchgrEcR* and *SchgrRXR*, were determined. The tissue distribution profile has been studied in the thoracic ganglia (TG), corpora cardiaca (CC), suboesophageal ganglion (SOG), salivary gland (Salgl), prothoracic glands (PG), fat body (Fb), midgut (MG), and ovaries (Ov) of 10-day-old female adult locusts and the testes and accessory glands (AG) of 10-day-old male adult locusts (Fig. 1A,B). The temporal distribution profile has been studied every other day throughout the first female reproductive cycle, starting on the day of moulting to the adult stage (Fig. 1C–H). For the temporal distribution profile, the ecdysteroid titre was also determined in the same animals.

Both components of the ecdysone receptor complex, *SchgrEcR* and *SchgrRXR*, show a wide tissue distribution profile (Fig. 1A,B). The highest transcript levels of *SchgrEcR* were found in the fat body of 10-day-old female adult locusts (Fig. 1A), while the highest transcript levels of *SchgrRXR* were found in the CA, the thoracic ganglia (TG) and the ovaries of 10-day-old female adult locusts, as well as in the testes of 10-day-old male adult locusts (Fig. 1B).

Since we wanted to investigate the role of the ecdysone receptor complex in female reproductive physiology, thereby looking at possible cross-talk with JH, the temporal distribution profiles of *SchgrEcR* and *SchgrRXR* were determined in the female ovary, fat body and corpora allata (CA) throughout the first reproductive cycle of female adult locusts (Fig. 1C–H). *SchgrEcR* transcript levels are high in the ovaries on the day of moulting to the adult stage, then decline and remain stable showing slightly higher transcript levels when ecdysteroid titres start rising (day 12) and as these decline again (day 18) (Fig. 1C). The *SchgrEcR* transcript levels in the fat body increase towards the end of the first reproductive cycle, which seems to coincide with the increase in ecdysteroid titre (Fig. 1D). In the CA the *SchgrEcR* transcript levels remain stable throughout the first reproductive cycle (Fig. 1E). As for *SchgrEcR*, *SchgrRXR* transcript levels in the ovaries are high at the day of moulting to the adult stage, after which they decline and remain stable throughout the rest of the first reproductive cycle (Fig. 1F). On the other hand, the temporal expression of *SchgrRXR* in the fat body and CA show a similar distribution profile, with high transcript levels at the day of moulting to the adult stage, a sudden decrease in 2-day-old female locusts and a peak right before the ecdysteroid titre rises (day 8–10) (Fig. 1G–H).

### RNA interference of the ecdysone receptor complex

#### Knockdown efficiency

RNAi-mediated knockdown of *SchgrEcR/SchgrRXR* resulted in significantly reduced transcript levels of both genes in all three tissues of interest. *SchgrEcR* transcript levels were 72%, 78% and 75% lower compared to the control animals in the ovaries, fat body and CA/CC complex respectively (Suppl. Fig. 1A–C). *SchgrRXR* transcript levels were significantly reduced with 40%, 81% and 59% in respectively the ovaries, fat body and CA/CC complex of *SchgrEcR/SchgrRXR* knockdown locusts when compared to control locusts (Suppl. Fig. 1D–F). We can therefore conclude that the knockdown of both components of the receptor complex was successful.

#### Effect on the female reproductive physiology

**Figure 2:**
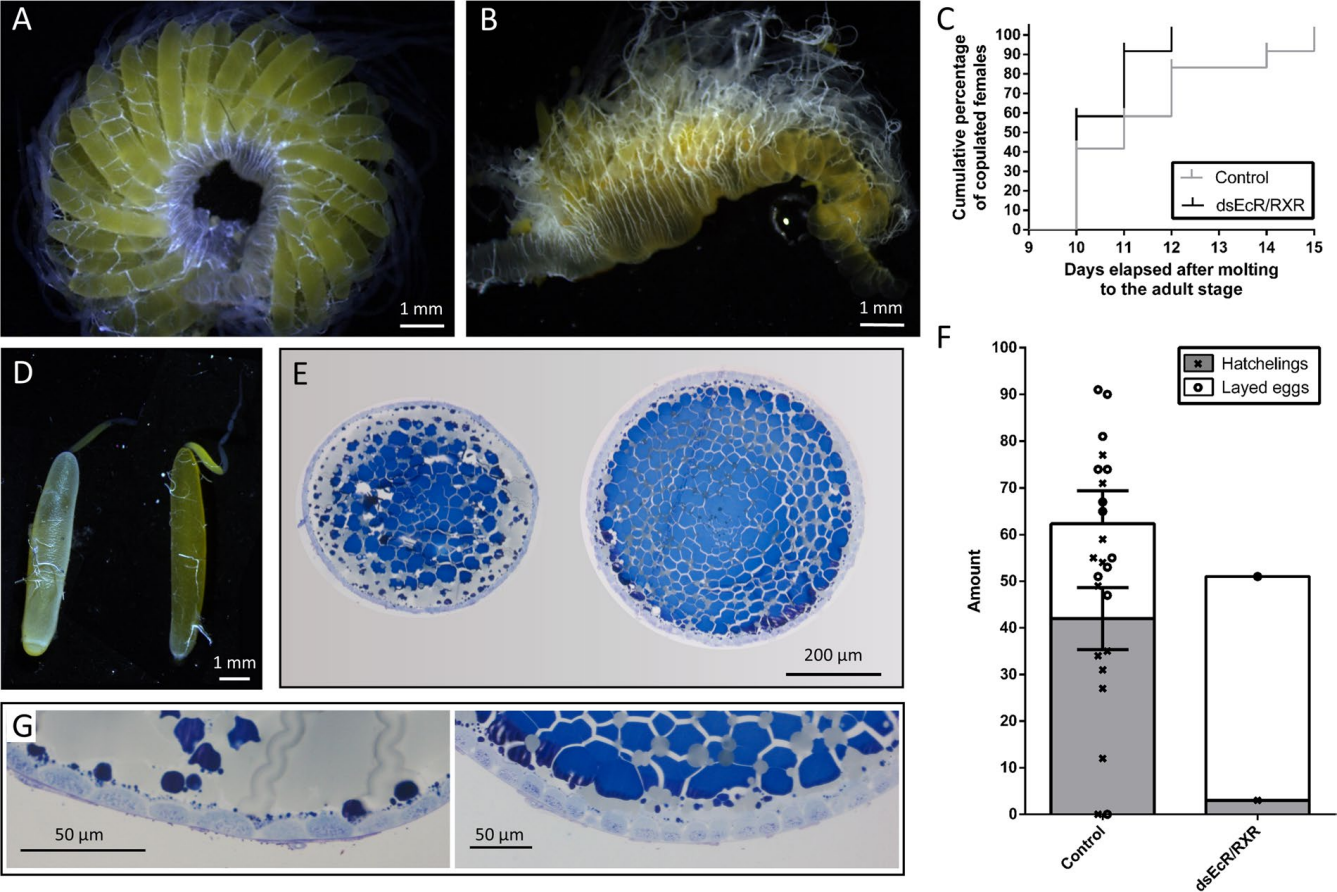
Effect of the RNAi-mediated knockdown of the ecdysone receptor complex (*SchgrEcR*/*SchgrRXR)* on the reproductive physiology of female *S. gregaria*. Locusts were injected as described in materials and methods. One part of the locusts (N = 15–20) was dissected on day 12 of the adult stage to assess ovary and oocyte growth (**A**,**B**,**D**,**E**,**G**), the other part of the locusts (N = 12) were kept alive to check copulation behaviour, fecundity and fertility (**C** and **F**). (**A**,**B**) Ovary of a representative (**A**) control locust (basal oocyte length = 3.1 mm) and (**B**) ds*EcR/RXR*-treated locusts (most basal oocytes resorbed, 2^nd^ cycle oocytes just started vitellogenesis and were about 2 mm in length). (**C**) Starting from day 10 of the adult stage the females were assayed for displaying mating behaviour, by allowing them to mate with mature virgin males. The cumulative percentage of females that mated is presented (N = 12). No significant differences can be observed (Log-rank (Mantel-Cox) test). (**D**) Ovarioles from control (left, basal oocyte length = 7 mm) and ds*EcR/RXR*-treated locusts (right, basal oocyte length = 7 mm). (**E**) Histological sections of the basal oocytes from a control locust (left, length = 3 mm – width = 0.5 mm) and a ds*EcR/RXR*-treated locust (right, length = 4.5 mm – width = 0.7 mm). (**F**) The number of eggs per egg pod was counted, as well as the number of hatchlings per egg pod. The data represent mean ± S.E.M. (bars), as well as the individual number of eggs (Ο) or hatchlings (X) per egg pod (N control = 12; only 1 out of 12 ds*EcR/RXR*-treated locusts laid eggs). (**G**) Magnification of the follicular cell layer of the basal oocytes shown in E (same order). Scale bars: A, B & D = 1 mm; E = 200 µm; G = 50 µm.

To investigate the effect of the RNAi-mediated knockdown of the ecdysone receptor complex on the female reproductive physiology, one group of locusts was dissected on day 12. Silencing *SchgrEcR/SchgrRXR* did not result in smaller oocytes. As long as the basal oocytes of *SchgrEcR/SchgrRXR* knockdown locusts did not reach the end of the vitellogenic stage, the ovaries had a normal appearance, resembling those of control locusts as shown in Fig. 2A. However, when the oocytes reached their full length of 7 mm, it appeared that the *SchgrEcR/SchgrRXR* locusts were not able to start choriogenesis. During normal development, as the oocyte enters the choriogenic stage of maturation, the egg shell or chorion is formed between the oocyte and the surrounding follicular cells, thereby giving the oocytes a shiny appearance (Fig. 2D left ovariole). After choriogenesis, ovulation occurs and the basal oocytes will pass from the ovarioles to the lateral oviducts. In the *SchgrEcR/SchgrRXR* knockdown locusts, this shiny appearance was never observed, even though the basal oocytes of several locusts reached their maximal length and should have entered the choriogenic stage (Fig. 2D right ovariole). Moreover, a lot of resorbing basal oocytes were observed in the ovaries of the *SchgrEcR/SchgrRXR* knockdown locusts that should have entered the choriogenic stage. Furthermore, throughout several experimental repeats, we never detected the presence of intact oocytes in the oviduct. Instead, a yellow substance resembling the content of vitellogenic oocytes was found in the oviduct and accessory glands, which was never seen in control locusts (Fig. 2B).

Transverse sections of vitellogenic terminal oocytes were examined to further investigate the observed phenotypes (Fig. 2E,G). The oocytes used for the sections shown in Fig. 2E both had a similar length (indicated in figure legend). In both control and *SchgrEcR/SchgrRXR* knockdown sections yolk material is visible (blue matrix), but less lipid droplets (greyish droplets) were present in the oocytes of *SchgrEcR/SchgrRXR* knockdown locusts when compared to those of control locusts. From the magnification of the follicular cell layer of these oocytes (Fig. 2G), we observed that the follicle cells surrounding the oocyte of *SchgrEcR/SchgrRXR* knockdown locusts were rather small and cubical, when compared to those of a control locust. It thus seems that the nuclear receptor knockdown interferes with the shape of the follicle cells surrounding the oocyte.

We also examined whether the treatments affected the first display of copulation behaviour, fecundity and fertility. Therefore, the timing of actual copulation with a virgin male, egg laying and hatching, as well as the numbers of eggs and percentage of hatchlings was determined. *SchgrEcR/SchgrRXR* knockdowns did not affect the timing of the first display of copulation behaviour (Fig. 2C). However, oviposition was severely impaired in the knockdown animals (Fig. 2F). Only one out of twelve *SchgrEcR/SchgrRXR* knockdown locusts successfully deposited her eggs in the sand/turf mixture. Two other *SchgrEcR/SchgrRXR* knockdown locusts tried to lay eggs, but only deposited a white/yellow foam against the side of the cage. All three females that attempted to oviposit did this around the same time as the control locusts. From the one successfully deposited egg pod only three locusts hatched, which then died within two days after hatching. It can be concluded that silencing *SchgrEcR/SchgrRXR* severely affects post-copulation events, and particularly oviposition.

#### Effect on ecdysteroid synthesis

We investigated if RNAi-mediated knockdown of the ecdysone receptor complex affected the ecdysteroid synthesis by measuring both the transcript levels of the known *Halloween* genes (Suppl. Fig. 2A–E), as well as the ecdysteroid titres in the haemolymph and the ecdysteroid levels in the developing ovaries (Suppl. Fig. 1F–H). Most of the ecdysteroids produced by the folliclar cell layer are incorporated in the growing oocytes as conjugates, while only a small fraction of the ecdysteroids is stored in the oocytes as ‘free’ ecdysteroids^42^. Moreover, a small fraction of the produced ecdysteroids will reach the haemolymph. The transcript levels of the known *Halloween* genes were not affected. Noteworthy is the high variation in the transcript levels of *SchgrSpo, SchgrPhm, SchgrSad* and *SchgrShd* after silencing *SchgrEcR/SchgrRXR*. Moreover, ecdysteroid titres in the haemolymph and ecdysteroid levels in the ovaries were not affected. It therefore seems that in adult female *S. gregaria* the *Schgr*EcR/*Schgr*RXR receptors do not induce a feedback on ecdysteroid synthesis.

#### Effect on JH synthesis and signalling

**Figure 3:**
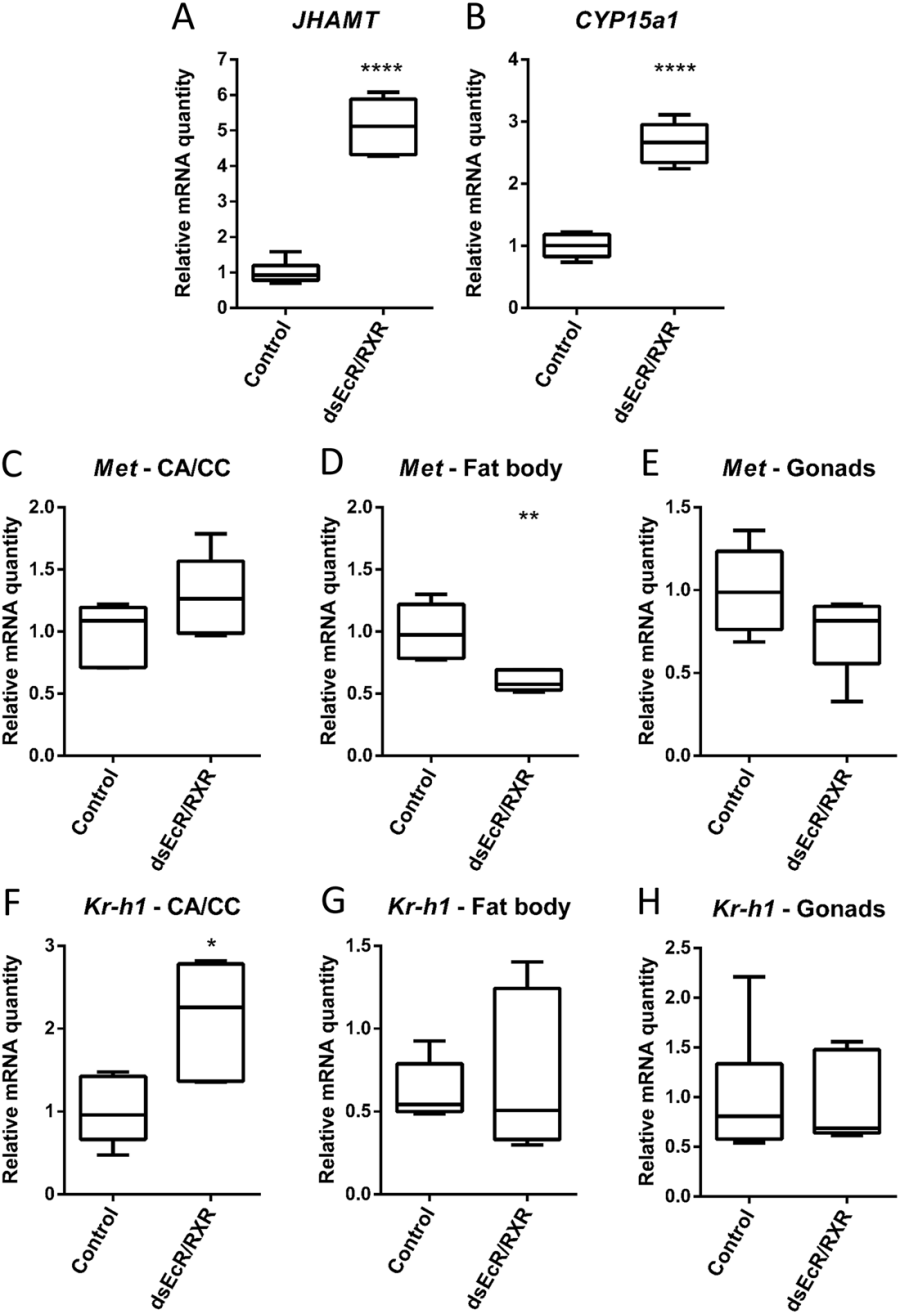
Effect of the RNAi-mediated knockdown of the ecdysone receptor complex (*SchgrEcR*/*SchgrRXR)* on JH synthesis and signalling in 12-day-old adult female *S. gregaria*. Locusts were injected as described in materials and methods and dissected on day 12 of the adult stage. Relative transcript levels of the last two enzymes involved in JH synthesis, *SchgrJHAMT* and *SchgrCYP15a1*, the JH receptor, *SchgrMet*, and a downstream response gene of JH, *SchgrKr-h1*, were measured in the CA/CC, fat body and ovaries of 12-day-old female locusts, using qRT-PCR. The data represent box plots (min to max) of five independent pools of three locusts, run in duplicate and normalized to *CG13220* and α-*tubulin1A* transcript levels for the ovaries, *CG13220, ubiquitin conjugating enzyme 10 (Ubi)* and *ribosomal protein 49* (*RP49)* transcript levels for the fat body, and *β-actin* and *EF1α* transcript levels for the CA/CC complex. Significant differences (p < 0.05, p < 0.01, and p < 0.0001) are indicated by (an) asterisk(s) (*, ** and **** respectively) (*t*-test or Mann-Withney U test on log-transformed data).

To investigate the possible cross-talk between *Schgr*EcR/SchgrRXR and JH synthesis and signalling, we checked the transcript levels of the genes encoding the enzymes responsible for the last two steps in the JH biosynthetic pathway (*SchgrJHAMT* = *juvenile hormone acid methyltransferase* and *SchgrCYP15A1* = *methyl farnesoate epoxidase*), the JH receptor methoprene-tolerant *(SchgrMet)*, as well as the JH response gene *Krüppel-homologue 1* (*SchgrKr-h1*) (Fig. 3). *SchgrJHAMT* and *SchgrCYP15a1* transcript levels were respectively 5.1 and 2.7 fold higher in *SchgrEcR/SchgrRXR* knockdown locusts compared to control locusts (Fig. 3A,B). The transcript levels of the JH receptor gene, *SchgrMet*, were significantly reduced with 35% in the fat body of *SchgrEcR/SchgrRXR* knockdown locusts (Fig. 3D), while no effect could be observed on the transcript levels of *Kr-h1* (Fig. 3G). *SchgrKr-h1* transcript levels were 2.1 fold higher in the CA/CC complex of ds*EcR/RXR*-treated locusts compared to control locusts (Fig. 3F). Transcript levels of neither *SchgrKr-h1* nor *SchgrMet* were affected in the ovaries of *SchgrEcR/SchgrRXR* knockdown locusts compared to control locusts (Fig. 3E,H). Also transcript levels of *SchgrMet* remained stable in the CA/CC complex upon treatment with dsRNA against *SchgrEcR/SchgrRXR* (Fig. 3C).

#### Effect on insulin-related peptide and neuroparsin signalling systems

**Figure 4:**
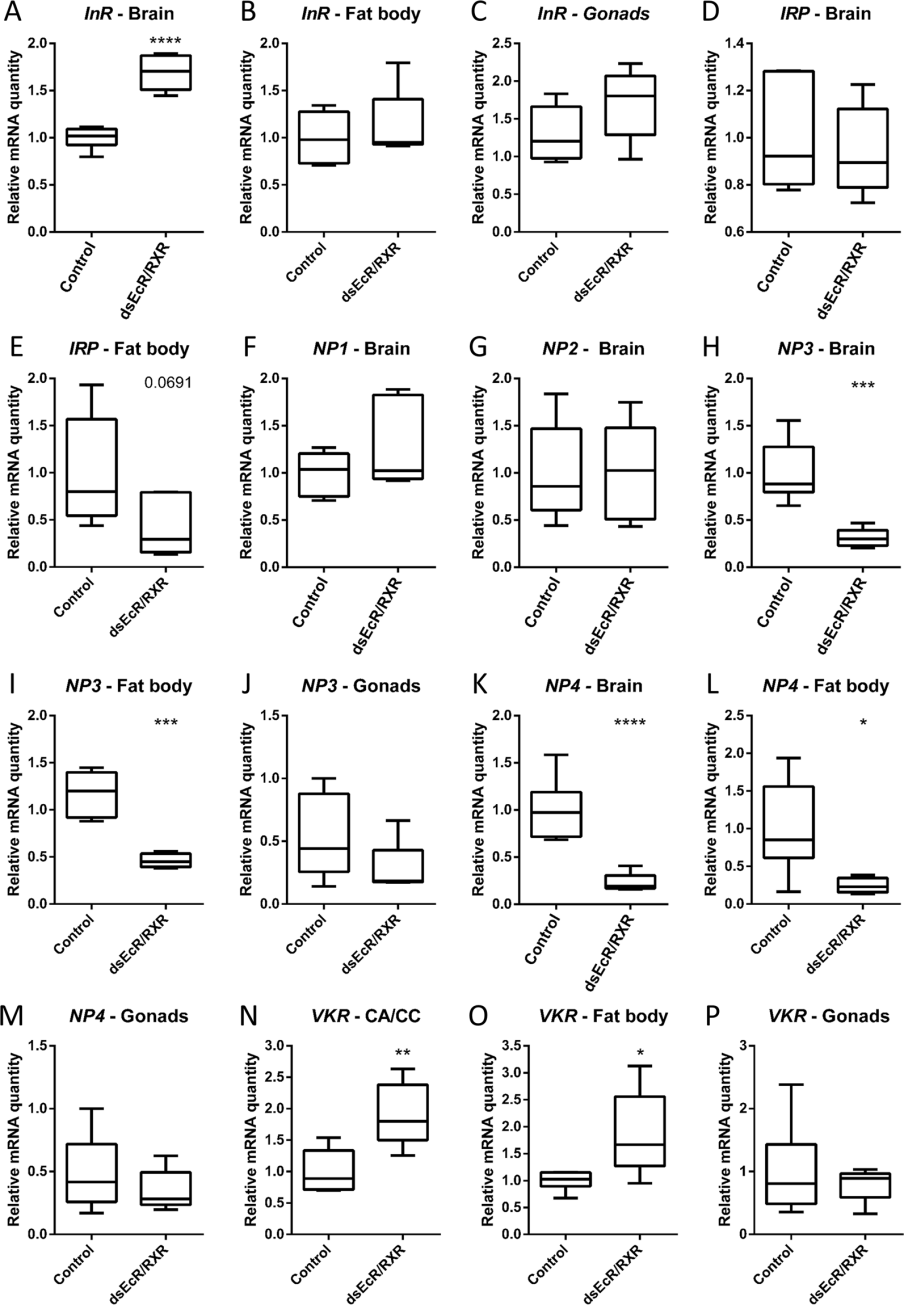
Effect of the RNAi-mediated knockdown of the ecdysone receptor complex (***SchgrEcR***/***SchgrRXR)*** on transcripts coding for the insulin receptor (InR), insulin-related peptide (IRP), neuroparsins (NP1–4) and the venus kinase receptor (VKR) in 12-day-old adult female *S. gregaria*. Locusts were injected as described in materials and methods and dissected on day 12 of the adult stage. Relative transcript levels of *SchgrInR*, *SchgrIRP, SchgrNP1-4* and *SchgrVKR* in the brain, fat body and ovaries of 12-day-old female locusts, using qRT-PCR. The data represent box plots (min to max) of five independent pools of three locusts, run in duplicate and normalized to *CG13220* and α-*tubulin1A* transcript levels for the ovaries, *CG13220, ubiquitin conjugating enzyme 10 (Ubi)* and *ribosomal protein 49* (*RP49)* transcript levels for the fat body, and *β-actin* and *EF1α* transcript levels for the brain. Significant differences (p < 0.05, p < 0.01, p < 0.001 and p < 0.0001) are indicated by (an) asterisk(s) (*, **, *** and **** respectively) (*t*-test or Mann-Withney U test on log-transformed data).

As insulin-related peptide (*Schgr*IRP) and neuroparsins (*Schgr*NPs) are known to have respectively gonadotropic and anti-gonadotropic roles in locusts, we investigated the possible cross-talk between *Schgr*EcR/*Schgr*RXR and these hormonal systems. To do so, we investigated the effect of the RNAi-mediated knockdown of *SchgrEcR*/*SchgrRXR* on the transcript levels of the insulin receptor (*SchgrInR)*, *SchgrIRP* and the known neuroparsins (*SchgrNP1*-*4*). Furthermore, we also investigated the effect of the knockdown on the transcript levels of the *venus kinase receptor* (*SchgrVKR)*, which is known in *A. aegypti* to act as the receptor for the neuroparsin-like ovary ecdysteroidogenic hormone^19^. If *SchgrVKR* acts as the receptor for NPs in locusts is still unknown. However, it does seem to play a role in the regulation of locust reproductive physiology^38^. The transcript levels of *SchgrInR* were significantly upregulated in the brain of *SchgrEcR/SchgrRXR* knockdown locusts when compared to control locusts (1.7 fold; Fig. 4A). However, *SchgrInR* transcript levels were not affected in the fat body and the ovaries (Fig. 4B,C). *SchgrIRP*, *SchgrNP1* and *SchgrNP2* transcript levels were also not affected (Fig. 4D–G). *SchgrNP3* and *SchgrNP4* transcript levels were, respectively, 69% and 77% lower in the brain of *SchgrEcR/SchgrRXR* locusts (Fig. 4H,K) and 31% and 36% lower in the fat body of *SchgrEcR/SchgrRXR* locusts, when compared to control locusts (Fig. 4I,L). No effect was observed on their transcript levels in the ovaries of *SchgrEcR/SchgrRXR* locusts (Fig. 4J,M). *SchgrVKR* transcript levels were 1.9 fold higher in both CA/CC and fat body of *SchgrEcR/SchgrRXR* locusts when compared to control locusts (Fig. 4N,O), while its transcript levels were not affected in the ovaries (Fig. 4P).

#### Effect on vitellogenin synthesis

We also investigated whether interfering with ecdysteroid signalling affected vitellogenin synthesis (Suppl. Fig. 3). For both known *vitellogenin* genes (*SchgrVg1* and *SchgrVg2*), the transcript levels were not significantly different from control locusts after silencing *SchgrEcR/SchgrRXR*.

## Discussion

### Expression patterns of *Schgr*EcR and *Schgr*RXR

The components of the ecdysone receptor complex, *SchgrEcR* and *SchgrRXR*, have previously been identified in *S. gregaria*^9^. The broad tissue distribution of *SchgrEcR* and *SchgrRXR* is consistent with findings in *P. americana, B. germanica* and *D. punctata* (Fig. 1A,B)^25,32,43^. The highest expression of *SchgrEcR* was observed in the fat body, a major player in energy storage and metabolism and the primary site of vitellogenin synthesis. An increase of *Schgr*EcR transcript levels was observed in fat body towards the end of the first reproductive cycle, which seemed to coincide with the observed peak of the ecdysteroid titre. The role of *SchgrEcR* in the female fat body remains unclear, since RNAi-mediated silencing of *SchgrEcR/SchgrRXR* did not affect vitellogenin transcript levels (Suppl. Fig. 3). This is contrary to *D. punctata* where ecdysteroid signalling was proven to be critical for the termination of vitellogenin synthesis^25^. It would be interesting to further investigate the role of *Schgr*EcR in the storage and release of energy reserves by the fat body, since it is known that in several insect species the nutritional status contributes at different levels to the control of the female reproductive physiology^44^. *SchgrRXR* was highly expressed in the ovaries, but the temporal distribution remained stable throughout the first reproductive cycle at the time points that were analysed. The significantly higher transcript levels in freshly moulted females compared to older females might be residual transcripts from the last nymphal stage. Both components of the ecdysone receptor complex were also highly expressed in the CA, indicating a possible role in the regulation of JH synthesis. We therefore determined the temporal distribution of both receptors in the CA (Fig. 1E,H). We observed a relatively stable expression of *SchgrEcR*, while the *SchgrRXR* transcript levels peak right before the ecdysteroid titre rises (day 8–10). This is contrary to *D. punctata*, where *DippuEcR* and *DippuRXR* transcript levels peak at the end of the first gonadotrophic cycle, coinciding with a drop in JH levels suggesting a feedback on JH biosynthesis^25^. The differences in expression profiles of *SchgrEcR* and *SchgrRXR* indicate possible distinct roles for both nuclear receptors, thereby possibly interacting with other, still unknown nuclear receptors. For instance, earlier studies have suggested that RXR/USP might function on its own, therefore binding different ligands^45^, such as retinoic acid and sesquiterpenoid ligands, like methyl farnesoate, a precursor of JH^46,47^.

### Ecdysteroid signalling complex is crucial in female *S. gregaria* reproductive physiology

In insects, ecdysteroids are incorporated into the growing oocytes as a maternal source during embryonic development^48^. This is also the case for *S. gregaria*, where ecdysteroids are mostly incorporated as polar conjugates of ecdysone or 20E^42^. Previous research has shown that the involvement of ecdysteroids in oogenesis is species-dependent (as reviewed by Swevers and Iatrou, 2009)^13^. Moreover, most recent research on the role of ecdysteroids in the female reproductive physiology focussed on the ecdysone receptor and its downstream response genes in the meroistic ovaries of Holometabola^13,49^. From an evolutionary perspective, it is therefore of great interest to further explore the role of ecdysteroids in the more ancestral panoistic type of ovaries as well.

Silencing the ecdysone receptor complex, *SchgrEcR/SchgrRXR*, had a significant impact on the female reproductive physiology in locusts. Ovulation and subsequent oviposition were blocked and a lot of resorption of the oocytes was observed upon silencing of the ecdysone receptor complex (Fig. 2). Our observations suggest that the ds*EcR/RXR*-treated locusts were not able to enter the choriogenic stage of oocyte maturation, resulting in more resorption compared to control locusts and part of the oocyte’s content leaking into the oviduct. It might also have been that those ovaries reached the point at which oocytes are normally passed from the ovarioles to the oviduct, and that the basal oocytes that did not resorb were sucked into the oviduct, resulting in the leakage of the oocyte’s content into the oviduct, due to the lack of a firm chorion which normally protects the oocytes upon ovulation. This observation is in accordance with previous research in *D. melanogaster, B. germanica* and *D. punctata*. In *D. melanogaster*, EcR and USP are known to regulate the transcription of chorion genes^50,51^ and mutants targeting the ecdysone receptor components resulted in thin and fragile eggshells^18,52,53^. In *B. germanica*, treatment with 20E causes precocious depositions of choriogenic material^27^. RNAi-mediated knockdown of *EcR/RXR* in *D. punctata* resulted in a similar phenotype, in which the basal oocytes reached their maximal length and subsequently were resorbed. Moreover, this research has proven that the RNAi-mediated knockdown of *EcR/RXR* reduced the expression of *FCP 3 C*, a gene which is selectively expressed in follicle cells during choriogenesis^25^. Since at present no chorion genes are known in *S. gregaria*, we were not able to verify whether the expression of any genes involved in locust choriogenesis was also affected by the ecdysone receptor complex knockdown.

### Cross-talk between ecdysteroids and other hormonal pathways

Silencing the ecdysone receptor complex did not influence ecdysteroid titres in the haemolymph or ecdysteroid levels in the ovaries. Remarkable, however, was the big variation in the transcript levels of *SchgrSpo, SchgrPhm, SchgrSad* and *SchgrShd* upon silencing of *SchgrEcR/SchgrRXR*. A possible explanation lies in the resorption of oocytes and big variation in the resulting oocyte sizes upon silencing of *SchgrEcR/SchgrRXR*. It is also important to keep in mind that we performed an RNAi-mediated knockdown of the receptor complex and as such, there might still be enough functional receptor present in order for ecdysteroids to feed back on their own synthesis. The observed results after silencing *SchgrEcR/SchgrRXR* in adult female locusts are also in contrast with previous research in *D. punctata*, where a knockdown of *EcR/RXR* resulted in significantly lower ecdysteroid titres in the haemolymph^25^.

We observed an upregulation of the last two JH synthesizing enzymes, as well as the JH response gene *Kr-h1* in the CA/CC of *SchgrEcR/SchgrRXR* knockdown locusts when compared to control locusts (Fig. 3). So it seems that the *Schgr*EcR/*Schgr*RXR receptors also regulate JH biosynthesis in vitellogenic female *S. gregaria*. However, the exact nature (direct or indirect) of this regulation is still unclear. Our findings are conform to the research done by Liu and co-workers^54^, where a cross-talk between ecdysteroids and juvenile hormones was observed in *D. melanogaster*. However, as *SchgrKr-h1* transcript levels were only higher in the CA/CC and not in the fat body and ovaries of *SchgrEcR/SchgrRXR* knockdown locusts, we hypothesize that those animals indeed synthesised more JH, but did not release it, or that JH was immediately metabolised upon release. The fact that *SchgrKr-h1* transcript levels were not affected in the fat body might also be due to significantly lower *SchgrMet* transcript levels in this tissue upon silencing of the ecdysone receptor complex. It therefore also seems that *SchgrMet* expression is under the control of the ecdysteroid receptor complex, but the exact nature of this control remains unclear. When comparing our results with those found after silencing *EcR/RXR* in adult female *D. punctata*^25^, some similarities and some differences can be observed. For instance, *DippuKr-h1* transcript levels were significantly lower in the CA/CC complex, but significantly higher in the fat body and ovary of *EcR/RXR* knockdown cockroaches. Moreover, *DippuMet* transcript levels were not affected in these three tissues. Furthermore, *DippuCYP15a1* transcript levels were significantly lower in *EcR/RXR* cockroaches, while the transcript levels of several other JH synthesis enzymes were significantly higher in these females. This recent research suggested both direct and indirect feedback of ecdysteroids on JH synthesis in *D. punctata*^25^. However, the exact reasons for the discrepancies observed between hemimetabolous insect species, when comparing our current data with those obtained with *D. punctata*^25^, remain unknown. More research is required to further explain this phylogenetic divergence. Moreover, the sensitivity of the CA to 20E seemed to be age-dependent^25^. Also, in *B. germanica*, it has been shown that ecdysteroids are responsible for lowering JH synthesis, which is needed for the termination of the gonadotrophic cycle^27^. As silencing of *Schgr*EcR/*Schgr*RXR resulted in the upregulation of the last to JH synthesizing enzymes in *S. gregaria*, we hypothesise that, similarly to *D. punctata* and *B. germanica*, high ecdysteroid titres at the end of the gonadotrophic cycle are needed to lower JH synthesis, subsequently initiate proper chorion formation, and thus terminate the gonadotrophic cycle in *S. gregaria*. However, this hypothesis deserves further attention in future studies. For instance, it might be interesting to investigate whether a rise in ecdysteroid titre earlier in the gonadotrophic cycle would result in a reduction of JH synthesis and consequently induction of early choriogenesis.

Next, we also investigated the possible cross-talk between *Schgr*EcR/*Schgr*RXR and insulin-related peptide or neuroparsin signalling systems, as these are known to exert gonadotropic and anti-gonadotropic actions, respectively^55^. The insulin signalling pathway (ISP) acts as a systemic nutrient sensor, thereby linking the insect’s nutritional state and reproductive processes. For an extensive review on this, the reader is referred to Badisco *et al*.^44^. No effect could be observed on the transcript levels of *SchgrIRP* upon silencing of *SchgrEcR/SchgrRXR* (Fig. 4), while the transcript levels of *SchgrInR* were significantly higher in the brain of *SchgrEcR/SchgrRXR* knockdown locusts (Fig. 4). As the transcript levels of *SchgrInR* were not affected in the fat body and the gonads, the cross-talk between *Schgr*EcR/*Schgr*RXR and the ISP seems to be tissue dependent. Moreover, we hypothesize that the observed role of *SchgrEcR/SchgrRXR* in the regulation of the ISP is most likely indirect. Our findings are in contrast with previous research in *B. mori* and *D. melanogaster*, where ecdysteroids were found to regulate the ISP by acting on the expression of the insulin-like peptides by the fat body^56–58^. The evolutionary distance between these holometabolous insects and our hemimetabolous research model, might explain these contrasting findings. However, further research is needed to unravel the exact molecular mechanisms that link ecdysteroid signalling and insulin signalling. The RNAi-mediated knockdown of *SchgrEcR/SchgrRXR* significantly reduced the transcript levels of *SchgrNP3* and *SchgrNP4* in the brain and fat body, but not in the ovaries (Fig. 4), which is another suggestion that ecdysteroids are able to exert tissue-specific actions. As a knockdown of *SchgrNPs* is known to affect vitellogenin levels in *S. gregaria*^55^, one might expect that vitellogenin levels would also be affected upon knockdown of *SchgrEcR/SchgrRXR* since this knockdown lowered the transcript levels of *SchgrNP3* and *SchgrNP4*. However, as this is not the case, *SchgrNP3* and *SchgrNP4* may not be the major neuroparsin variants that are involved in the control of vitellogenin expression. Alternatively, their effects may have been compensated by other changes that were directly or indirectly induced by *Schgr*EcR/*Schgr*RXR knockdown.

## Conclusions

Ecdysteroid signalling is crucial for successful ovulation and oviposition in *S. gregaria*, since RNAi-mediated silencing of the ecdysone receptor complex affects ovarian maturation by affecting choriogenesis. We have also found evidence for a cross-talk between ecdysteroid signalling and other important hormonal pathways in adult female *S. gregaria*. For instance, *Schgr*EcR/*Schgr*RXR silencing influences the expression of JH biosynthesis and signalling components in the CA. However, the RNAi-mediated knockdown of the ecdysone receptor complex might not affect JH titres in the rest of the body, as *SchgrKr-h1* transcript levels were not affected in the fat body and the ovaries. Furthermore, we have also shown an *in vivo* effect of the ecdysone receptor complex knockdown on the tissue specific regulation of *SchgrInR, SchgrNP3* and *SchgrNP4* gene expression.

## Electronic supplementary material

Supplementary Figures and Tables
