# Supplementary material for: The ecdysone receptor complex is essential for the reproductive success in the female desert locust, Schistocerca gregaria
Source: Sci Rep. 2019 Jan 9;9:15. doi: 10.1038/s41598-018-36763-9 (PMC6327042; doi:10.1038/s41598-018-36763-9)
Supplement: Supplementary file 1 — Supplementary Figures and Tables [file 41598_2018_36763_MOESM1_ESM.pdf]

The ecdysone receptor complex is essential for the reproductive success in the female desert locust, *Schistocerca gregaria*

Cynthia Lenaerts[1], Elisabeth Marchal[1], Paulien Peeters[1], Jozef Vanden Broeck[1]

[1]Molecular and Developmental Physiology and Signal Transduction research group, KU Leuven, Naamsestraat 59, P.O. Box 02465, B-3000 Leuven, Belgium.

Correspondence: Dr. Cynthia Lenaerts, Molecular and Developmental Physiology and Signal Transduction research group, KU Leuven, Naamsestraat 59, P.O. Box 02465, B-3000 Leuven, Belgium. E-mail: cynthia.lenaerts@kuleuven.be.

Supplementary figure 1

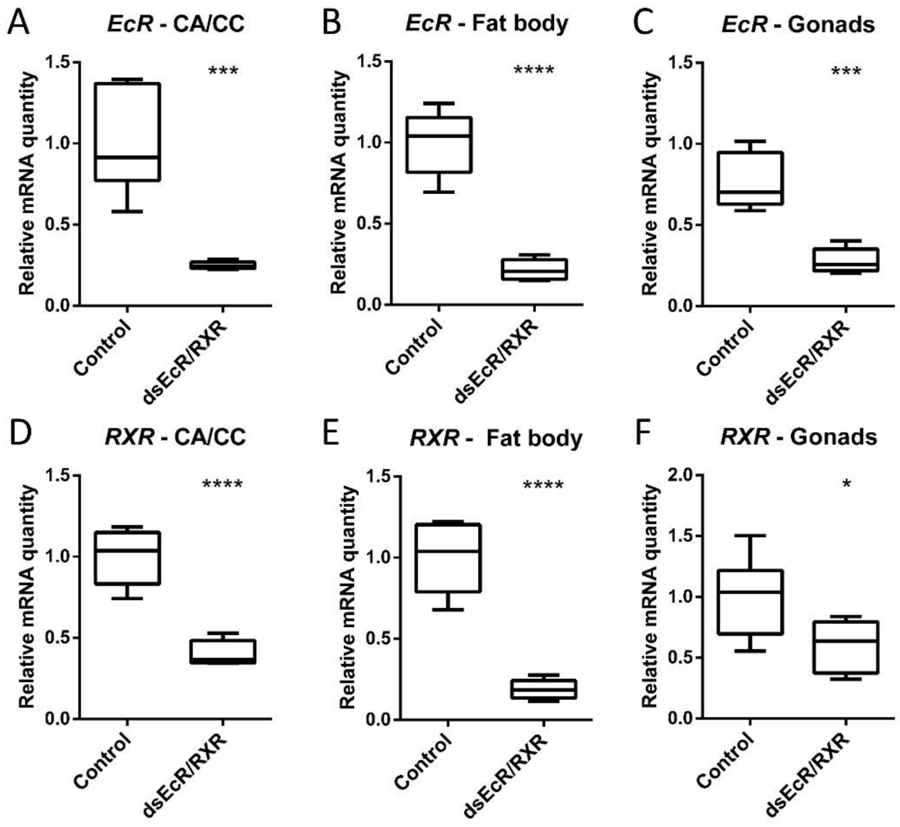

Supplementary figure 1: Efficiency of the RNAi-mediated knockdown of the ecdysone receptor complex (*SchgrEcR/SchgrRXR)* in 12-day-old adult female *S. gregaria*. Locusts were injected as described in materials and methods and dissected on day 12 of the adult stage. Relative transcript levels of (A-C) *SchgrEcR* and (D-F) *SchgrRXR* were measured in (A/D) the CA/CC complex, (B/E) the fat body and (C/F) the ovaries of 12-day-old female locusts, using qRT-PCR. The data represent box plots (min to max) of five independent pools of three locusts, run in duplicate and normalized to *CG13220* and α-*tubulin1A* transcript levels for the ovaries, *CG13220, ubiquitin conjugating enzyme 10 (Ubi)* and *ribosomal protein 49* (*RP49)* transcript levels for the fat body, and *β-actin* and *EF1α* transcript levels for the CA/CC complex. Significant differences ($p < 0.05$, $p < 0.001$ and $p < 0.0001$) are indicated by (an) asterisk(s) (*, *** and **** respectively) (two-sided Welch's *t*-test on log-transformed data).

Supplementary figure 2

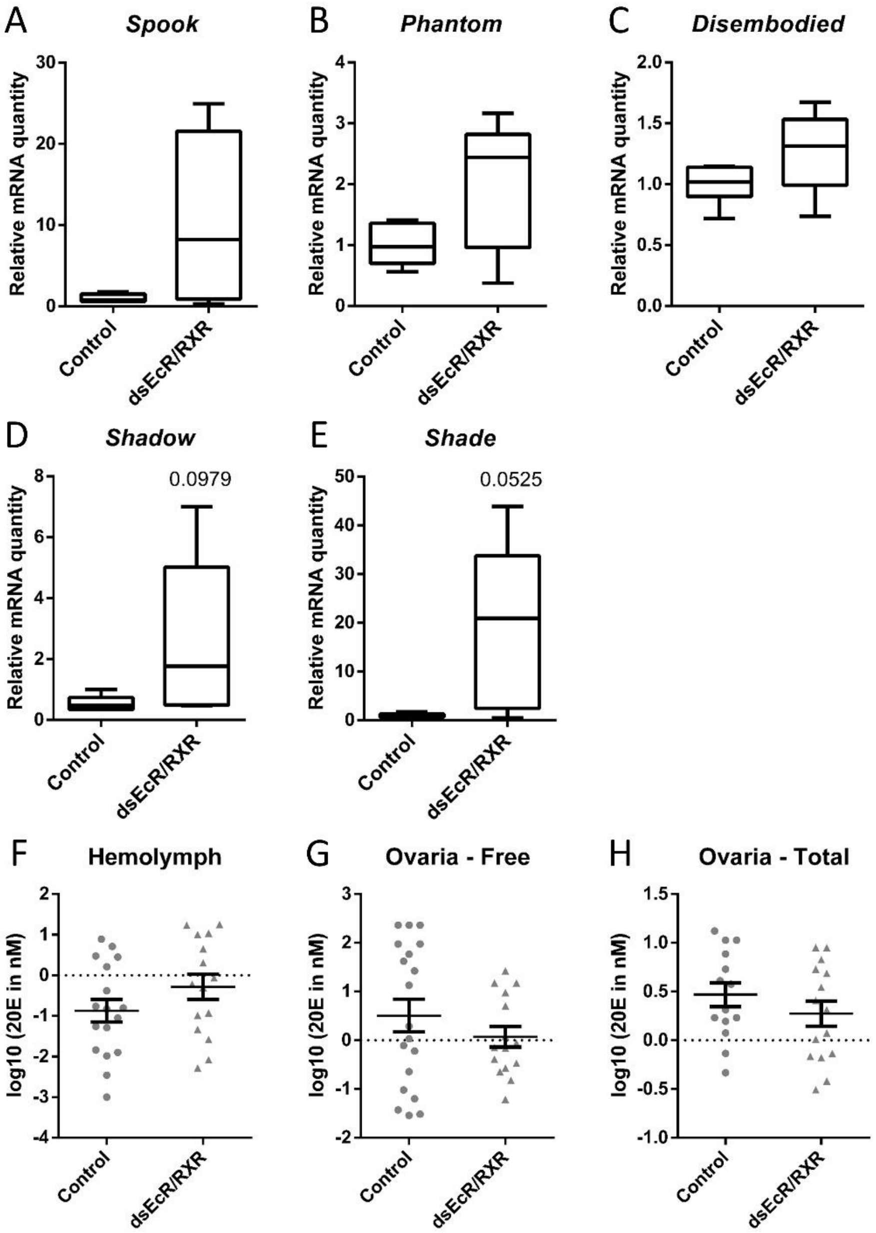

Supplementary figure 2: Effect of RNAi-mediated knockdown of the ecdysone receptor complex (*SchgrEcR/SchgrRXR)* on ecdysteroid synthesis in 12-day-old adult female *S. gregaria*. Locusts were injected as described in materials and methods and dissected on day 12 of the adult stage. (A-E) Relative transcript levels of the *Halloween* genes, *SchgrSpo*, *SchgrPhm*, *SchgrDib, SchgrSad* and *SchgrShd* were measured in the ovaries of 12-day-old control and ds*EcR/RXR*-treated adult *S. gregaria*, using qRT-PCR. The data represent box plots (min to max) of five independent pools of three locusts, run in duplicate and normalized to *CG13220* and α-*tubulin1A* transcript levels. (F-G) Ecdysteroid titres in the haemolymph and free and total ecdysteroid levels in the ovaries of 12-day-old control and ds*EcR/RXR*-treated female locusts. Ecdysteroid titres, expressed in ng 20E equivalents per µL haemolymph, and ecdysteroid levels, expressed in ng 20E equivalents per ovary, were measured with an EIA. The data represent mean ± S.E.M. of individual animals (n = 15-19), as well as the individual values (grey dots). No significant differences were observed.

Supplementary figure 3

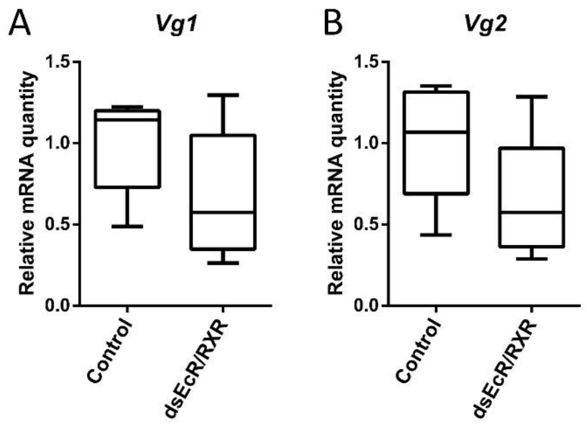

Supplementary figure 3: Effect of RNAi-mediated knockdown of the ecdysone receptor complex (*SchgrEcR*/*SchgrRXR)* on vitellogenin synthesis in 12-day-old adult female *S. gregaria*. Locusts were injected as described in materials and methods and dissected on day 12 of the adult stage. Relative transcript levels of *SchgrVg1* and *SchgrVg2* were measured in the fat body of 12-day-old control and ds*EcR/RXR*-treated adult *S. gregaria*, using qRT-PCR. The data represent box plots (min to max) of five independent pools of three locusts, run in duplicate and normalized to *CG13220, ubiquitin conjugating enzyme 10 (Ubi)* and *ribosomal protein 49* (*RP49)* transcript levels.

Supplementary table 1

Oligonucleotide sequences for primers used in qRT-PCR.

Reference genes	Forward primer	Reverse primer
α-tubulin1A	5'-TGACAATGAGGCCATCTATG-3'	5'-TGCTTCCATACCCAGGAATGA-3'
CG13220	5'-TGTTCAGTTTTGGCTCTGTTCTGA-3'	5'-ACTGTTCTCCGGCAGAATGC-3'
Ubi	5'-GACTTTGAGGTGTGGCGTAG-3'	5'-GGATCACAAACACAGAACGA-3'
RP49	5'-CGCTACAAGAAGCTTAAGAGGTCAT-3'	5'-CCTACGGCGCACTCTGTTG-3'
β-actin	5'-AATTACCATTGGTAACGAGCGATT-3'	5'-TGCTTCCATACCCAGGAATGA-3'
EF1α	5'-GATGCTCCAGGCCACAGAGA-3'	5'-TGCACAGTCGGCCTGTGAT-3'

Target genes	Forward primer	Reverse primer
SchgrEcR	5'-AAGGTTGATAATGCGGAATATGC-3'	5'-GTGATGGGCGCTCTGAAAAT-3'
SchgrRXR	5'-AATGCCTCGCTATGGGAATG-3'	5'-TCCTTTGTTCGCTGCCTTTC-3'
SchgrE74	5'-AAAGGAAGAGCCGTGAAG-3'	5'-CCCTGTTCGTCCACTTAAT-3'
SchgrE75	5'-GCACACCAACAGCATGATCT-3'	5'-ACCACCGAGCAGAACAGC-3'
SchgrBr-C	5'-CAGATCATCAGCACCACAATTTACA-3'	5'-CGTGCCCGTTTGTTCGTT-3'
SchgrHR3	5'-CACCAGTACAGGCAATGA-3'	5'-TCAGTTGCTAGCTCAAGAC-3'
SchgrFTZ-F1	5'-CCTACTCTAGCCGATCACTTCAATG-3'	5'-CAAATGTAATCGCTGAGGTCAAAT-3'
SchgrJHAMT	5'-CGGAGCAAAGGCAAGCA-3'	5'-CCACTTCACCGCCTGGTTT-3'
SchgrCYP15a1	5'-AAAGCAACTTCATCATTCACAGATG-3'	5'-CAGAGCCAGCCATGAACAAA-3'
SchgrMet	5'-GGTGCCTGAAGAGGAAGAAA-3'	5'-ATGGAGGTGATGAAGGAGAAAG-3'
SchgrKr-h1	5'-CTCCAAGACGTTCATCCAGAG-3'	5'-TGCTTGGAGCAGGTGAAG-3'
SchgrInR	5'TGGATGGCACCAGAAAGCTT-3'	5'-CACAGCACTACACCGTAGCT-3'
SchgrIRP	5'-CCGTGGCAACTACAACACCAT-3'	5'-TCCGCGTCCGACACATCT-3'
SchgrNP1	5'-CGCACGGACACGTCAACA-3'	5'-GTGACACGTAGGCGAAAGGAA-3'
SchgrNP2	5'-CGCTGGCGGTGACACA-3'	5'-CAAACTCCACTGTACAAAATAAATAGACTAGA-3'
SchgrNP3	5'-GCGGAATCGGCATGGA-3'	5'-TCACAGAGCAACCGGAACATT-3'
SchgrNP4	5'-TGGCGACTCTCCAGTGCTT-3'	5'-TGACACATTCATTCCTCTTCTGACA-3'
SchgrVKR	5'-GCATCTTGGCATTGATTTGCTA-3'	5'-GGAATCTCCCATTTGTCAAGAGTT-3'
SchgrSpo	5'-CAACATCTTCACCAGCTACATGTG-3'	5'-GGGTCGTCGTAGTCGAAGGA-3'
SchgrPhm	5'-CGCAGAGCCCGGACAAC-3'	5'-CGAACATGTCGGCCATGA-3'
SchgrDib	5'-CCCAGGCTGCTATCGAGACT -3'	5'-CGACGACCAGGCCTATGTAGTT -3'
SchgrSad	5'-ATCGTGGCCGAGATTACGAA -3'	5'-AGCACCATCTCCGGATCCT -3'
SchgrShd	5'-CCGCCGTCATTGACTTCATA-3'	5'-GTGAGCTCCCAAGCGTGG-3'
SchgrVg1	5'-CCGCTGAACATCACTGCAAT-3'	5'-ACTTGGGCCAAATGGATGAG-3'
SchgrVg2	5'-GCTACCCGCAATCTGTAAAATACA-3'	5'-CGACTGTGAAAGGGCATTGA-3'

Abbreviations: *Ubi = ubiquitin conjugating enzyme 10, RP49 = ribosomal protein 49, EF1α = elongation factor 1 alpha, EcR = ecdysone receptor, RXR = retinoid-X-receptor, Br-C = Broad-Complex, FTZ-F1 = fushi tarazu factor 1, JHAMT = juvenile hormone acid methyltransferase, CYP15a1 = methyl farnesoate epoxidase, Kr-h1 = Krüppel-homolog 1, Met = methoprene-tolerant receptor, InR = insuline receptor, IRP = insuline related peptide, NP = neuroparsin, VKR = venus kinase receptor, Spo = spook, Phm = phantom, Dib = disembodied, Sad = shadow, Shd = shade, Vg = vitellogenin*

Supplementary table 2

Oligonucleotide sequences for primers used in dsRNA construct design. Underlined sequences are the T7 promoter sequences.

Target genes	Forward primer	Reverse primer
SchgrEcR	5’-GAAATTAATACGACTCACTATAGGGCC<br>ACGTGAGGTTTCGGCACATC-3’	5’-GAAATTAATACGACTCACTATAGGGCC<br>GTTTCCCCCATACCAGCCAG-3’
SchgrRXR	5’-GAAATTAATACGACTCACTATAGGGCC<br>GCTCAATGGGTCCACAGTCA-3’	5’-GAAATTAATACGACTCACTATAGGGCC<br>ACACCATAATGCTTCCCGCT-3’
GFP	5’-TAATACGACTCACTATAGGGAGA<br>AAGGTGATGCTACATACGGAA-3’	5’-TAATACGACTCACTATAGGGAGA<br>ATCCCAGCAGCAGTTACAAAC-3’

Abbreviations: *EcR = ecdysone receptor, RXR = retinoid-X-receptor, GFP = green fluorescent protein*
